# Leukocyte redistribution as immunological biomarker of corticosteroid resistance in severe asthma

**DOI:** 10.1111/cea.14128

**Published:** 2022-04-01

**Authors:** Carlos Cardoso‐Vigueros, Tobias von Blumenthal, Beate Rückert, Arturo O. Rinaldi, Ge Tan, Anita Dreher, Urszula Radzikowska, Günter Menz, Peter Schmid‐Grendelmeier, Cezmi A. Akdis, Milena Sokolowska

**Affiliations:** ^1^ Department of Allergy University Hospital of Zurich Zurich Switzerland; ^2^ Swiss Institute of Allergy and Asthma Research (SIAF) University of Zurich Davos Switzerland; ^3^ Christine Kühne – Center for Allergy Research and Education (CK‐CARE) Davos Switzerland; ^4^ Hochgebirgsklinik Davos (HGK) Davos Switzerland

**Keywords:** asthma phenotypes, biological therapy, corticosteroids resistance, leukocyte redistribution, severe asthma, treatment asthma

## Abstract

**Background:**

Earlier studies have suggested that the leukocyte redistribution can be considered as an immunological marker of the clinical response to corticosteroids (CS), representing an easy measurable potential biomarker in severe asthma.

**Objective:**

The aim of this study was to determinate the utility of the leukocyte redistribution as a biomarker of disease heterogeneity in patients with severe asthma and as a bioindicator of potential CS resistance.

**Methods:**

We developed an unbiased clustering approach based on the clinical data and the flow cytometry results of peripheral blood leukocyte phenotypes of 142 patients with severe asthma before and after systemic CS administration.

**Results:**

Based on the differences in the blood count eosinophils, neutrophils and lymphocytes, together with the flow cytometry measurements of basic T cell, B cell and NK cell subpopulations before and after systemic CS administration, we identified two severe asthma clusters, which differed in the cell frequencies, response to CS and atopy status. Patients in cluster 1 had higher frequency of blood eosinophils at baseline, were sensitized to less allergens and had better steroid responsiveness, measured as the pronounced leukocyte redistribution after the administration of systemic CS. Patients in cluster 2 were determined by the higher frequency of B‐cells and stronger IgE sensitization status to the multiple allergens. They also displayed higher steroid resistance, as the clinical correlate for the lower leukocyte redistribution after administration of systemic CS.

**Conclusion:**

The flow cytometry‐based profiling of the basic populations of immune cells in the blood and its analysis before and after systemic corticosteroid administration could improve personalized treatment approaches in patients with severe asthma.

## Graphical abstract

Intravenous challenge with corticosteroids identified two clusters of patients with severe asthma, who had different leukocyte redistribution as analysed by the complete blood count and flow cytometry. Unbiased clinical phenotyping after clustering revealed that Cluster 1 and 2 patients varied slightly at baseline in clinical characteristics and in the need for systemic corticosteroids. LR might potentially serve as a biomarker of corticosteroid sensitivity to identify the biological reserve of the immune system. FEV1, forced expiratory volume in 1 s; FVC, forced vital capacity; ICS, inhaled corticosteroids; LR, leucocyte redistribution; OCS, oral corticosteroids. Created with www.biorender.com.

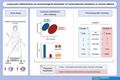

**Key messages**

- Cellular response to intravenous corticosteroids, so called leukocyte redistribution, identified two clusters of severe asthma patients
- Post‐clustering phenotyping revealed differences in these patients’ baseline cell frequency, lung function, atopy and oral corticosteroids use
- Leukocyte redistribution in response to intravenous corticosteroids might be a biomarker of steroid resistance

## 1 INTRODUCTION

Corticosteroids (CS) are the most effective anti‐inflammatory drugs used in the treatment of asthma. However, 5–10% of asthma patients respond poorly to inhaled CS (ICS), requiring very high doses of ICS, the usage of oral CS, other immunosuppressants or biologics to achieve asthma control.^1^ The mechanisms of CS resistance in asthma are not well understood, but several cellular and molecular events underlying this phenomenon have been suggested. They include abnormalities of glucocorticoid receptor and downstream signalling pathways, altered pro‐inflammatory cytokines’ expression and associated epigenetic changes.^2^, ^3^, ^4^ These events have been strongly related to the genetic and environmental factors such as cigarette smoking, exposure to moulds or respiratory infections, leading to the abnormal behaviour and function of immune responses.^5^, ^6^, ^7^, ^8^ The phenomenon of CS resistance occurs in patients with severe asthma, characterized in addition by frequent exacerbations and often profound impairment in the lung function.^9^ Severe asthma is associated with increased morbidity, mortality and increased healthcare burden. Moreover, the side effects caused by an excessive use of steroids dramatically impact patients’ quality of life. Therefore, an early identification of patients with CS resistance is very important to avoid long‐lasting administration of high doses of steroids and associated side effects, and to determine early initiation of the alternative therapies.

Currently, the targeted selection of such patients is difficult, because the diagnosis of severe asthma is usually based on the symptoms and the intensity of the treatment.^10^ Moreover, severe asthma is a complex, heterogeneous disease with a wide spectrum of underlying pathogenic mechanisms (so called endotypes), subsequent inflammatory phenotypes and variable response to the treatment.^10^, ^11^, ^12^, ^13^, ^14^ In addition, to date, there is no established objective biomarker for determining steroid resistance in patients with asthma in the clinical settings. Such biomarker should be easy to determine, inexpensive and ideally should enable further phenotyping and endotyping for the determination of a specific therapy with biologicals.^15^, ^16^, ^17^

Systemic CS treatment induces an immediate redistribution of peripheral blood leukocytes.^18^, ^19^ This still incompletely understood phenomenon, sometimes called leukocyte redistribution (LR), refers to a temporary decrease in the frequency of lymphocytes and an increase in natural killer (NK) cells and granulocytes in the peripheral blood.^19^, ^20^ Such changes are also observed under strenuous exercise or other stress‐inducing conditions, which lead to a short‐term increase of endogenous CS levels.^21^, ^22^ Interestingly, the observed alterations are profoundly immunosuppressive, since the migratory and phagocytic capacities of granulocytes are decreased.^23^, ^24^ The peak of this redistribution in healthy individuals is observed in the first 4 h following steroid administration and it usually returns to normal levels within 24 h.^19^, ^25^ Earlier studies suggested that the acute LR, to some extent might reflect sensitivity to CSs in patients with asthma and the efficacy of these drugs.^26^ Karagiannidis et al. proposed to determine immunological sensitivity to CS by means of flow cytometry, allowing for an immunological categorization of difficult to treat asthma patients.^26^ However, no further characterization of these patients has been done so far and the unbiased assessment of the value of LR has not been performed.

Therefore, the aim of this study was to determinate the utility of the LR as a biomarker of disease heterogeneity in patients with severe asthma and a potential bioindicator of CS resistance. For this purpose, we developed an unbiased clustering approach based on the combined blood count and the flow cytometry results of peripheral blood cells to determinate the LR after systemic CS administration in order to reflect the pathophysiologic processes and disease heterogeneity in patients with severe asthma.

## 2 MATERIAL AND METHODS

### 2.1 Patients

One hundred forty‐two patients with severe asthma participating at a rehabilitation program at the Hochgebirgsklinik of Davos (Switzerland) between 2000 and 2017 were studied. All patients fulfilled the criteria of severe disease according to the ATS/ERS criteria guidelines for severe asthma.^9^ Moreover, most of the patients had repeated hospitalizations because of recurrent exacerbations. At the time of investigations, all patients obtained therapy according to the Global Initiative for Asthma (GINA) guidelines of that time (http://www.ginaasthma.org).

Lung function tests were performed with the patients in a sitting position according to the guidelines of the American Thoracic Society/European Respiratory Society 2005.^27^ Dynamic tidal volumes (Forced expiratory volume in 1 s [FEV1], forced vital capacity [FVC] and FEV1/FVC) were determined using a pneumotachograph spirometer (Masterlab‐Compact Labor, CareFusion, Viasys Healthcare). Measurements of Fractional exhaled nitric oxide (FeNO) were performed according the ATS/ERS Recommendations for Standardized Procedures for the measurement of exhaled lower respiratory nitric oxide 2005^28^ through the chemiluminescence detection method (Analyzer CLD 88 Sp/Spiroware). The Skin prick testing (SPT) was performed according to the European standards^29^ with a standard prick test panel for the most common aeroallergens in Central Europe. Measurement of total serum IgE was carried out using a fully automated system (ImmunoCAP Thermo Fisher Scientific), according to the manufacturer's protocol. The doses of ICS were calculated as the budesonide equivalent. Reported ICS were taken regularly by the patients at baseline on admission to the hospital. Reported oral CS (OCS) were taken by the patients on admission to the hospital to aid in disease control. Patients’ characteristics are shown in Table 1. The ethical permission has been received from the Ethical Committee of Canton of Zurich (BASEC‐Nr. 2019‐00734) and all patients gave an informed consent.

**TABLE 1:** Participants’ demographic and asthma phenotypic characteristics before stratification

	All patients (*n* = 142)
Age (year)	45 (19–74)
Sex, female/male	104/38
Weight (kg)	80 (44–145)
Height (cm)	170 (144–197)
Obesity (body mass index >35), yes/no	37/102
Active smoking history (1 pack‐year or more), yes/no	44/91
Atopy, yes/no	102/40
Aeroallergens sensitization (number of aeroallergens)	3 (0–18)
Sensitivity to aspirin (history), yes/no	34/105
Nasal polyps, yes/no	49/92
Dose of inhaled budesonide equivalent (μg)	1727 (0–5600)
Dose of oral corticosteroids (mg)	27 (0–90)
FEV_1_ (L)	1.86 (0.58–3.9)
FEV_1_ (%)	57.6 (11.7–98.3)
FEV_1_/FVC (%)	71.13 (41.42–100)
FeNO (ppb) on admission	58 (5–310)
Serum IgE level (IU/ml)	378 (0–5000)

Note

Data are presented as median and range. FEV_1_ – forced expiratory volume in 1 s. FVC – forced vital capacity. FeNO‐fraction of exhaled nitric oxide.

### 2.2 Intervention

Each patient received intravenous administration of an average dose of 50 mg of prednisolone in the beginning of hospitalization. Blood was collected before intravenous prednisolone administration at 07:30 am and again 3 h after the intervention.

### 2.3 Blood count and flow cytometry

EDTA blood was analysed by the automated Sysmex system to determine the frequencies of basic blood cell types including lymphocytes, eosinophils and neutrophils. For the flow cytometry, EDTA blood was stained with the following antibodies: CYTO‐STAT tetraCHROME CD45‐FITC/CD4‐RD1/CD8‐ECD/CD3‐PC5, CYTO‐STAT tetraCHROME CD45‐FITC/CD56‐RD1/CD19‐ECD/CD3‐PC5 (Beckman Coulter), anti‐CD16‐PE (3G8, BD Bioscience), CD25‐RD1 (ACT‐1, Dako‐Agilent), HLC‐DR‐ECD (IO/Immu‐357, Beckman Coulter) and CD23‐RD1 (MHM6, Dako‐Agilent). After 15‐min incubation at room temperature in the dark, the samples were lysed which was followed by the paraformaldehyde fixation using the Coulter Q‐Prep Workstation with IMMUNOPREP reagent system (Beckman Coulter). Isotype controls had the equal protein concentration as the test antibodies. Four‐colours flow cytometry was preformed using an EPICS™ XL‐MCL (Beckman Coulter) using the software Expo™ 32 version for data acquisition and evaluation.

### 2.4 Bioinformatic and statistical analysis

Unsupervised clustering of patients was performed based on the differences of the seven flow cytometry parameters (frequency of CD3^+^ cells, CD4^+^ cells, CD8^+^ cells, NK cells, natural killer T (NKT) cells, B cells and the CD4^+^/CD8^+^ ratio) and three parameters from the blood count (lymphocytes, eosinophils and neutrophils) before and after the intravenous GC administration. As a first step, we examined the clustering tendency of the data by Hopkins’ statistic, showing that the data is highly clustered. Next, the Silhouette method suggested that the optimal number of clusters was two. Then, K‐means clustering approach was used to partition the patients into two groups.

Mann–Whitney *U* test was used to compare differences between two different unrelated groups, such as patients from Cluster 1 and those from Cluster 2. Wilcoxon signed rank test was used to compare differences between paired groups, such as the same patient in “before” and “after” conditions. All statistical analyses were performed with GraphPad Prism 8.3 (GraphPad Software). A *p*‐value of <.05 was considered statistically significant.

## 3 RESULTS

### 3.1 Intravenous injection of CS reveals two clusters of severe asthma patients, based on the differences in their leukocyte redistribution

**FIGURE 1:**
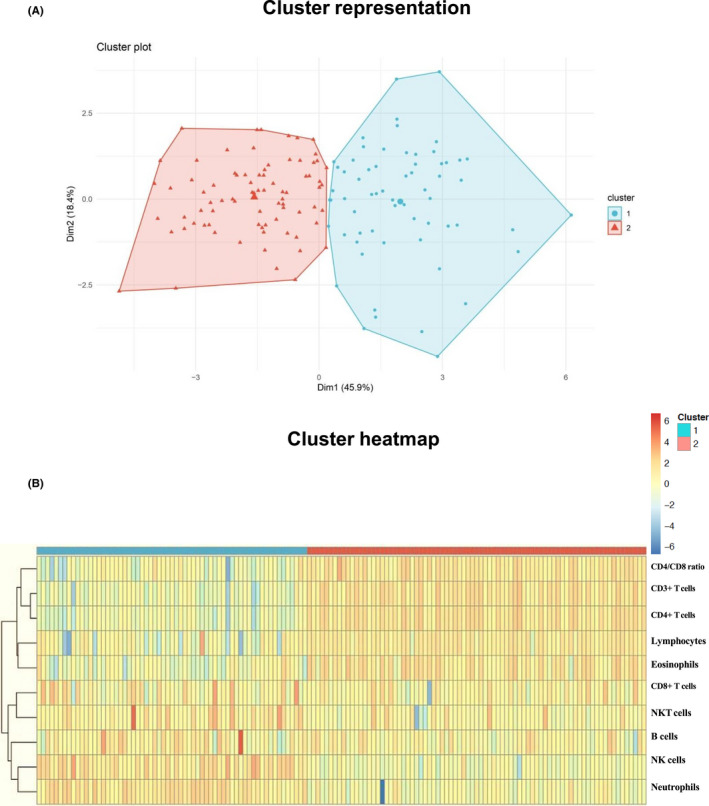
Intravenous injection with steroids reveals two clusters of severe asthma patients

In order to determine differences in the response to systemic CS treatment in patients with severe asthma, we analysed the behaviour of ten cell populations and parameters: neutrophils, eosinophils, total lymphocytes, CD3^+^ T cells, CD4^+^ T cells, CD8^+^ T cells, NK cells, NKT cells, B cells and the CD4^+^/CD8^+^ ratio before and after intravenous treatment with CS. Based on the fold change in the cell frequency and using the unsupervised clustering approach outlined in the Methods, we identified two groups of patients who responded differently to CS administration (Figure 1A). Cluster 1 corresponded to the smaller group of patients (*n* = 63; 44.36% of subjects). In these patients, intravenous application of CS led to a more intensive redistribution of leukocytes. There was a marked increase in neutrophils, NK cells, NKT and B cells and a profound decrease in eosinophils, lymphocytes, CD3^+^ T cells and CD4^+^ T cells and in the CD4^+^/CD8^+^ ratio (Figure 1B). Cluster 2 corresponded to the slightly larger group of patients in our severe asthma cohort (*n* = 79; 55.63% of subjects). The administration of systemic CS in these patients led to a moderate redistribution of leukocytes compared with the patients in Cluster 1 as shown in the clusters’ heatmap (Figure 1B). There was only a slight increase in the frequency of neutrophils, NK cells and NKT cells in this cluster. The frequency of B cells remained almost unchanged. There was also a moderate decrease in eosinophils, lymphocytes, CD3^+^ T cells and CD4^+^ T cells and in the CD4^+^/CD8^+^ ratio. CD8^+^ T cells remained unchanged in both groups.

### 3.2 Two clusters of patients vary at baseline in the frequency of eosinophils, lymphocytes and neutrophils, which are further differently influenced by intravenous CS administration

**FIGURE 2:**
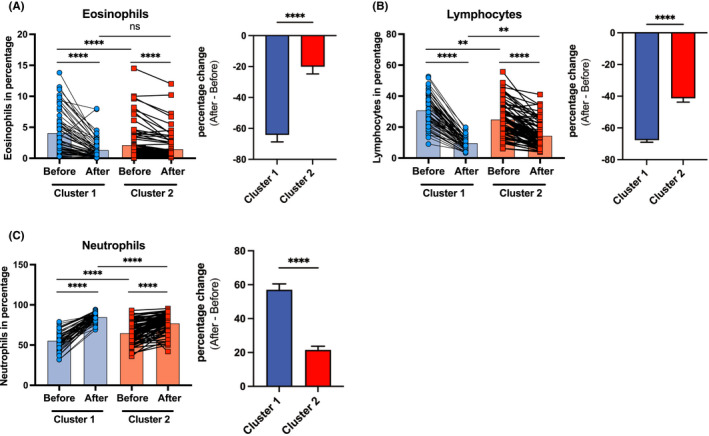
Cluster 1 and 2 patients with severe asthma differ at baseline in the frequency of eosinophils, lymphocytes and neutrophils, as well as in the magnitude of leukocyte redistribution after GC treatment

Next, we analysed in detail the baseline and treatment‐initiated alterations in the basic cellular composition, revealed by the blood count, in these two identified clusters of patients. At baseline, Cluster 1 patients had higher frequency of eosinophils (Figure 2A, left panel) and lymphocytes (Figure 2B, left panel) than patients from Cluster 2. In contrast, Cluster 2 patients had higher frequency of neutrophils (Figure 2C, left panel).

Importantly, the magnitude of effects of CS administration (percentage change of after vs before) was significantly different in each of the clusters, whereas the direction of changes was similar (Table 2, Figure 2A–C, right panels). Eosinophils showed a decrease of 64.20% in Cluster 1 [from 4.11% (SD 3.74) to 1.36% (SD 1.62)] and a decrease of 20.10% in Cluster 2 [from 2.16% (SD 2.82) to 1.51% (SD 2.19)], leading to the similar eosinophil percentage in both clusters after intervention (Figure 2A and Table 2). The percentage change in Cluster 1 was significantly bigger than in Cluster 2, suggesting that the exposure of these patients to the pharmacological levels of CS induced a stronger migration of the eosinophils out of circulation probably due to the already higher eosinophil levels in the periphery.

**TABLE 2:** Leukocyte redistribution after intravenous CS administration

Cell type	Cluster 1			Cluster 2			*p*‐value
	Before (%)	After (%)	Percentage change	Before (%)	After (%)	Percentage change	
Eosinophils	4.11	1.36	−64.20	2.16	1.51	−20.10	<.0001
Lymphocytes	30.98	9.79	−67.71	25.09	14.56	−41.31	<.0001
Neutrophils	55.85	85.00	57.00	65.11	77.34	21.48	<.0001
NK cells	10.69	25.29	169.62	14.04	16.92	39.77	<.0001
NKT cells	2.21	3.77	70.86	2.91	3.29	23.06	<.0001
B cells	8.54	10.57	26.80	23.66	23.59	−1.26	<.0001
CD3^+^ cells	80.43	62.99	−21.90	74.67	70.31	−5.58	<.0001
CD4^+^ cells	55.67	38.86	30.72	47.49	43.30	−8.46	<.0001
CD8^+^ cells	23.67	23.59	−1.26	26.03	25.52	−2.11	.6010

Note

For the different cell types the percentage mean calculated before and after the steroid administration are reported, together with the mean of the corresponding percentage changes. The last column reports the *p*‐value calculated by Mann–Whitney *U* test comparing the percentage changes of patients of Cluster 1 and those of Cluster 2.

Lymphocytes behaved in a similar fashion, presenting significantly bigger change induced by the systemic administration of CS in Cluster 1 patients. The percentage of lymphocytes decreased by 67.71% in Cluster 1 [from 30.98% (SD 9.8) to 9.79% (SD 3.82)] and by 41.31% in Cluster 2 [from 25.09% (SD 10.64) to 14.56% (SD 8.37)]. Interestingly, whereas at baseline Cluster 1 patients had higher frequency of lymphocytes, it reversed after CS administration with Cluster 1 patients having significantly lower percentage of lymphocytes than Cluster 2 patients (Figure 2B and Table 2).

CS significantly increased the percentage of neutrophils 3 h after intravenous prednisolone administration in both clusters of patients. Again, the magnitudes of changes were bigger in Cluster 1 patients. The number percentage of neutrophils showed an increase of 57.00% in Cluster 1 [from 55.85% (SD 10.5) to 85.00% (SD 5.4)] and an increase of only 21.48% in Cluster 2 [from 65.11% (SD 5.41) to 77.34% (SD 11.8)] (Table 2). Notably, when at baseline, Cluster 1 patients had lower frequency of neutrophils than Cluster 2 patients, it changed significantly after the CS treatment (Figure 2C).

### 3.3 Two clusters of patients vary at baseline in CD3+ T cells, CD4+ T cells, B cells and NK cells, as well as in the response of these populations to CS administration

**FIGURE 3:**
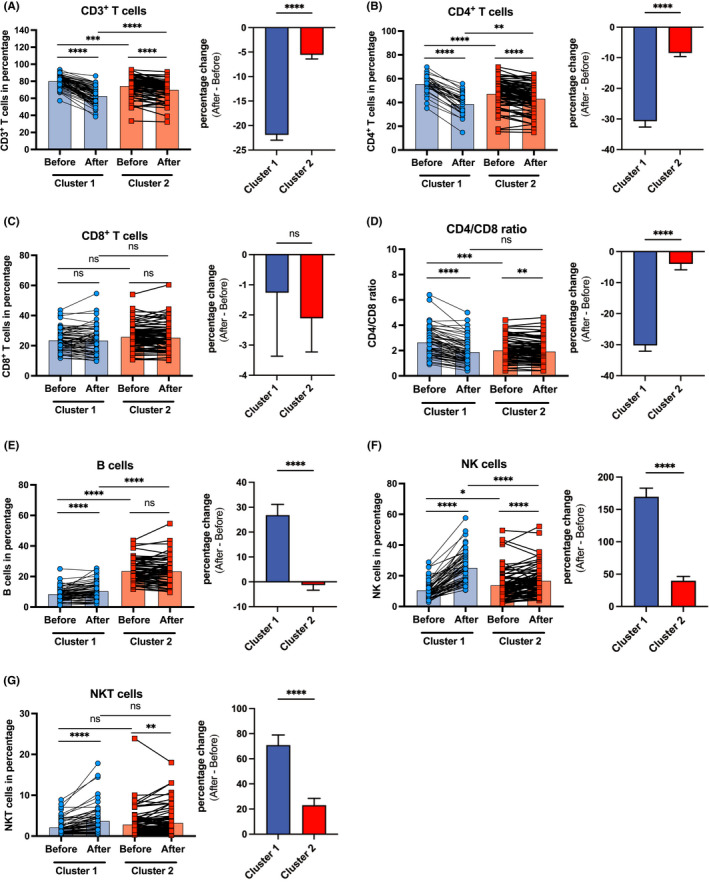
Cluster 1 and Cluster 2 patients with severe asthma differ at baseline in the frequency of CD3^+^ T cells, CD4^+^ T cells, B cells, NK cells and in the response of these populations to CS administration

Next, we analysed in more detail the baseline and CS treatment‐initiated changes in the subpopulations of lymphocytes by flow cytometry. We found that Cluster 1 patients had higher baseline frequency and absolute numbers of CD3^+^ T cells, CD4^+^ T cells, but not CD8^+^ T cells (Figure 3A–C; Figure S1A–C). They also had higher CD4^+^/CD8^+^ T cell ratio at baseline (Figure 3D). In addition, Cluster 1 patients had lower baseline frequency, and a trend to lower absolute numbers of B cells and NK cells (Figure 3E,F; Figure S1D,E).

The frequency of CD3^+^ T cells, CD4^+^ T cells and their absolute numbers, as well as the CD4^+^ /CD8^+^ ratio decreased significantly in both clusters of patients following CS administration (Figure 3A,B,D; Figure S1A,B). Again, the magnitudes of these decreases were more pronounced in Cluster 1 patients [frequency of CD3^+^ T cells changed from 80.43% (SD 6.9) to 62.9% (SD 10.01), frequency of CD4^+^ T cells from 55.67% (SD 8.75) to 38.86% (SD 9.33) and the CD4^+^/CD8^+^ ratio from 2.66 (SD 1.11) to 1.9 (SD 0.98)], in contrast to that in Cluster 2 [frequency of CD3^+^ cells from 74.67% (SD 11.31) to 70.31% (SD 10.77), frequency of CD4^+^ cells from 47.49% (SD 11.07) to 43.30% (SD 10.79) and the CD4^+^/CD8^+^ ratio from 2.04 (SD 0.86) to 1.94 (SD 0.86)] (Figure 3A–C, Table 2). Interestingly, the percentage of CD8^+^ T cells remained unchanged after CS administration in both groups of patients, even if they dropped significantly in absolute numbers (Figure 3C; Figure S1C, Table 2).

Notably, 3 h after intravenous prednisolone administration, there was a slight but significant increase in the percentage of circulating B cells only in Cluster 1 patients (Figure 3E, Table 2), even if the B‐cell numbers significantly decreased in both clusters [frequency of B cells changed from 226.5 (SD 146.8) to 103.6 (SD 65.23) in Cluster 1, and from 235.8 (SD 177.2) to 154.4 (SD 103.5) in Cluster 2] (Figure S1D). The percentage of B cells in Cluster 2 remained high and showed no further significant change after the CS administration (Figure 3E, Table 2). Importantly, after the intervention, there was significantly more B cells in Cluster 2 patients also measured in absolute numbers (Figure S1D).

Finally, the frequency of NK cells and NKT cells increased more markedly in Cluster 1 after CS administration (Figure 3F,G, Table 2), even if their absolute numbers decreased in Cluster 2 (Figure S1H) or in both clusters (Figure S1I). In detail, NK cells showed an increase of 169.62% in Cluster 1 and 39.77% in Cluster 2, whereas NKT cells showed an increase of 70.86% in Cluster 1 and 23.06% in Cluster 2 (Table 2).

### 3.4 Cluster 1 and 2 patients with severe asthma differ in asthma phenotypic characteristics

Having found that patients from Cluster 1 and 2 differed in terms of the baseline and treatment‐induced responses in several cell populations in the peripheral blood, we analysed if there were any differences in their demographics and asthma phenotype parameters. There were no differences in the age, height, weight and gender between these the two clusters of patients (Table 3). Looking at the asthma phenotypic characteristics no significant difference could be noted in terms of sensitivity to aspirin, smoking history and other underlying medical conditions or parameters such as nasal polyps or FeNO (Table 3).

**TABLE 3:** Demographic and asthma phenotypic characteristics at baseline after stratification into Cluster 1 and Cluster 2

	Cluster 1 (*n* = 63)	Cluster 2 (*n* = 79)	*p* value
Age (year)	47 (19–74)	42 (23–69)	.113
Sex, female/male	45/18	59/20	.663
Weight (kg)	74.3 (46–136)	79.8 (44–145)	.179
Height (cm)	168 (144–197)	169.5 (150–193)	.447
Obesity (body mass index >35), yes/no	13/48	24/54	.211
Active smoking history (1 pack‐year or more), yes/no	21/40	23/51	.679
Aeroallergens sensitization (number of aeroallergens)	2 (0–11)	3 (0–18)	.045*
Sensitivity to aspirin (history), yes/no	12/49	22/56	.24
Nasal polyps, yes/no	21/42	28/50	.75
Dose of inhaled budesonide equivalent (μg)	1600 (320–5600)	1600 (0–4800)	.941
Dose of oral corticosteroids (mg)	20 (0–80)	25 (0–90)	.043*
FEV_1_ (L)	1.54 (0.58–3.9)	1.91 (0.67–3.48)	.264
FEV_1_ (%)	57.8 (22.1–98.3)	57.9 (11.7–96.8)	.784
FVC/FEV_1_ (%)	68.41 (43.3–92.14)	71.60 (41.42–100)	.006*
FeNO (ppb) on admission	41.5 (6–289)	35 (5–310)	.391
Serum IgE level (IU/ml)	104 (0–1159)	107 (2.75–5000)	.011*

Note

Data are presented as median and range. FEV_1_ – forced expiratory volume in 1 second. FVC – forced vital capacity. FeNO‐fraction of exhaled nitric oxide. Data were analyzed by t‐test, Mann–Whitney *U* test or Fisher exact test. Bold values indicates *p* less than .05.

**FIGURE 4:**
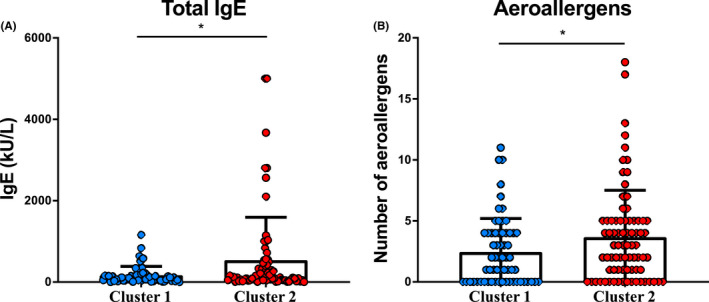
Cluster 2 patients showed higher baseline total IgE levels and aeroallergen sensitization

Interestingly though, we found that patients from these two clusters differed significantly in terms of total IgE, number of aeroallergens they were sensitive to, FEV1/FVC and the doses of systemic steroids (Figure 4 and Table 3). Patients from Cluster 1 had lower levels of total IgE (Figure 4A) and were sensitive to lower number of allergens (Figure 4B). They were also treated with lower doses of systemic CSs, even if they had lower FEV1/FVC (Table 3).

## 4 DISCUSSION

In this study, by applying an unsupervised clustering approach, we identified two clusters of severe asthma patients. These patients differed in the extent of peripheral blood leukocyte and lymphocyte redistributions in response to the intravenous treatment with CSs. Moreover, in the post‐clustering analysis, we found that these patients also differed in some clinical and inflammatory features, indicating that they may have divergent endotype of severe asthma, and thus, may benefit from different treatment.

Multiple cluster analyses in various asthma cohorts have identified several clinical phenotypes of adult asthma in an overlapping way, suggesting that the phenotypes might be fairly generalizable across different patient populations.^30^, ^31^, ^32^, ^33^, ^34^, ^35^, ^36^ The heterogeneity of severe asthma has been extensively studied in detail in the big consortia including SARP and U‐Biopred in the US and Europe. These studies revealed that patients with severe asthma had more symptoms compared to patients with mild/moderate disease, with worse quality of life and frequent exacerbations associated with persistent eosinophilic inflammation in peripheral blood, sputum and bronchoalveolar lavage despite high doses of systemic CS. They also had a higher incidence of nasal polyps and gastro‐oesophageal reflux with lower lung function.^37^

The phenotype of asthma in patients from our cohort corresponded in general to the most severe phenotype previously described with frequent exacerbations, the worse mean FEV1 (FEV1 (57.6%) and a T2‐high profile, despite treatment with OCS.^38^ Patients with this profile are the most common participants included in the asthma studies for novel T2‐inflammation focused therapeutics, for example, anti‐IL‐13 and anti‐IL‐5 monoclonal antibodies.^39^, ^40^, ^41^, ^42^ Blood eosinophil count is used as the hallmark of these T2‐high endotype.^43^ However, it is able to predict therapeutic responses to CS or biologicals only in about two thirds of the cases.^16^, ^44^, ^45^, ^46^ This may in part be due to the wider heterogeneity of T2 phenotypes and because peripheral eosinophilia may not fully reflect the T2 cytokine and receptor activity.^33^, ^47^, ^48^, ^49^ In agreement with these observations, we could distinguish two groups reacting differently to the systemic CSs administration in our severe asthma cohort.

Cluster 1 patients had higher baseline eosinophilia, despite lower total IgE, being sensitized to the lower number of aeroallergens and lower FEV1/FVC than Cluster 2 patients. Interestingly, the frequency and numbers of lymphocytes, mainly T cells (CD3^+^ T cells) of T helper phenotype (CD4^+^ T) was also higher, whereas the B‐cell frequency was lower in those patients at baseline in comparison with Cluster 2 patients. Moreover, the intensity of leukocyte and lymphocyte redistribution was more pronounced in these patients with a marked decrease of eosinophils, total lymphocytes, CD3^+^ T cells, CD4^+^ T cells and CD4^+^/CD8^+^ ratio and a notable increase of neutrophils, NK cells and NKT cells after CS administration. Importantly, patients in this cluster needed at baseline lower doses of systemic CS to control the disease. Altogether, our findings of prominent leukocyte and lymphocyte redistribution, higher baseline eosinophils and Th cells, but lower frequency of B cells, lower total IgE and less enhanced atopic status may be consistent with a better clinical response of these patients to the treatment with steroids (eosinophilic and steroid responder phenotype). This cluster slightly corresponds to the previously described severe late‐onset eosinophilic asthma, but not all patients from this cluster would fit to this description, based on the baseline eosinophil count, lung function, FeNO, sensitization and most importantly the response to CS.^50^ Their higher responsiveness to CS in the prednisone intervention might suggest their better response to CS, although it needs to be determined in the future.

In contrast, Cluster 2 patients who responded to the intravenous CSs treatment in the less pronounced manner, had higher total IgE levels and were sensitized to a larger number of aeroallergens, and had slightly higher FEV1/FVC. In agreement with the higher total IgE, the percentage and absolute numbers of circulating B cells were also higher in these patients at baseline or after CSs administration. In addition, patients in Cluster 2 had to be treated with significantly higher doses of systemic CSs to achieve asthma control or have not achieved asthma control despite higher systemic steroid doses as compared to the patients in Cluster 1, which might correspond to the higher neutrophils counts at baseline. This multiallergic phenotype with high total IgE, and high B‐cell frequency might correspond to the steroid resistance in this patient group (B‐cell associated and steroid non‐responder phenotype). This phenotype of severe asthma resembles to some extent the early‐onset allergic asthma phenotype with increased Th2 cytokines and atopy reported in various phenotyping studies,^51^, ^52^ yet it is interesting that those patients in the present study can be also characterized by the higher neutrophils, B‐cell levels, lower LR and higher needs for systemic steroid.

Several candidate biomarkers for asthma heterogeneity have been investigated.^53^, ^54^ Until now, only sputum eosinophils, blood eosinophil count, FeNO measurement, total IgE, specific IgE and the SPT have been recommended for diagnosis, phenotyping and prognosis for treatment with ICS and biologics in the international asthma guidelines.^41^, ^55^, ^56^ There are also a few studies about the biomarkers of response to CS in severe asthma. Sputum eosinophils and FeNO were the best predictors of a favourable response to oral prednisolone in severe asthmatics.^57^ LR after prednisone intervention might, therefore, serve as an additional bioindicator of CS sensitivity in the most severe asthma patients to identify the biological reserve of the immune system and perhaps predict the clinical response to CS, but it needs to be prospectively tested. In patients with a B‐cell associated, steroid non‐responder phenotype (weak LR after CS administration), a further increase in CS doses may be unnecessary and other therapeutic options such as biologics can be considered at an early stage. This kind of patients may potentially benefit more from anti‐IgE or anti‐IL4Rα therapy. It has been shown that anti‐IgE therapy works better in severe asthma patients with high total IgE, who cannot be controlled with systemic CSs.^58^ Anti‐IL4Ra therapy can also be useful in blocking IgE production by the plasma cells y,^59^ which would be presumably more frequent in patients with higher frequency of CD20^+^ B cells, as in case of Cluster 2 patients. In contrast, patients with eosinophilic, steroid responder phenotype (Cluster 1 patients with strong LR after CS administration) may still benefit from the systemic CSs therapy. In addition, anti‐IL‐5 therapies could be very advantageous in such patients, in order to minimize systemic CSs side effects.

**FIGURE 5:**
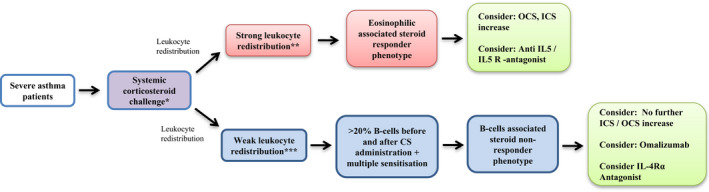
Medical hypothesis: proposed 2‐step algorithm to identify two clusters of patients with severe asthma based on the leukocyte redistribution trial after systemic CS administration

Due to the relatively easy clinical implementation of the LR method present in this study, we believe that it would be possible to translate this clustering approach and the basic blood count and flow cytometric parameters used for this into the real‐world severe asthma treatment (Figure 5). As can be seen in the present study, a clinical flow cytometry with only four colours in addition to the blood count, would be sufficient to identify CS resistant patients. This novel biomarker could improve clinical and research strategies, allowing the prediction of minimal steroid doses with maximal treatment success and the better selection of patients requiring alternative therapies and the selection of biological therapies based on the underlying immunological response. However, prospective studies are needed to confirm the utility of this approach in predicting clinical treatment responses.

Our study has a few limitations. The sample size was relatively small and contained only one cohort of patients from a single centre. In addition, we were unable to perform repetitive measurements of the LR during the study period, which prevents us to determine the stability of this biomarker over time or to test the effects of clinical intervention on the cellular frequencies and LR in the prospective manner. New prospective studies are required to determine whether LR can be used as a biomarker to predict the therapeutic response to CS and various biologicals in severe asthma and to evaluate the advantages of the LR in addition to the current biomarkers in patients with severe asthma.

## CONFLICT OF INTEREST

CC reported grants from GSK, honoraria from AstraZeneca and GSK. PS reports honoraria from GSK, Novartis and Sanofi. CA reported research grants from Allergopharma, Idorsia, Swiss National Science Foundation, Christine Kühne‐Center for Allergy Research and Education, European Commission's Horison's 2020 Framework Programme, Cure, Novartis Research Institutes, AstraZeneca and SciBase, advisory board of Sanofi/Regeneron, GSK and Novartis, consulting fees from Novartis. MS reported research grants from Swiss National Science Foundation, Novartis and GSK and speaker's fee from AstraZeneca and a leadership in the European Academy of Allergy and Clinical Immunology: Secretary of the Board of the Basic and Clinical Immunology Section. Other authors did not report any conflict of interest.

## AUTHOR CONTRIBUTIONS

CCV* and TVB* were involved in experiment design, data collection/analysis, data interpretation, manuscript preparation and review; BR involved in data collection/analysis and manuscript review; AR and AD were involved in data analysis and manuscript review; GT and UR were involved in data analysis, manuscript preparation and review; GM was involved in experiment design, data collection/analysis and manuscript review; PSG was involved in data interpretation and manuscript review; CA was involved in experimental design, data interpretation and manuscript review; MS was involved in experiment design, data collection/analysis, data interpretation, manuscript preparation and review. *Co‐first authors, both contributed equally.

## ETHICAL APPROVAL

The ethical permission has been received from the Ethical Committee of Canton of Zurich (BASEC‐Nr. 2019‐00734) and all patients gave an informed consent.

## Supporting information

Fig S1
