# Supplementary material for: Leukocyte redistribution as immunological biomarker of corticosteroid resistance in severe asthma
Source: Clin Exp Allergy. 2022 Apr 1;52(10):1183–94. doi: 10.1111/cea.14128 (PMC9790739; doi:10.1111/cea.14128)
Supplement: Supplementary file 1 — Fig S1 [file CEA-52-1183-s001.pdf]

Supplementary Figure 1

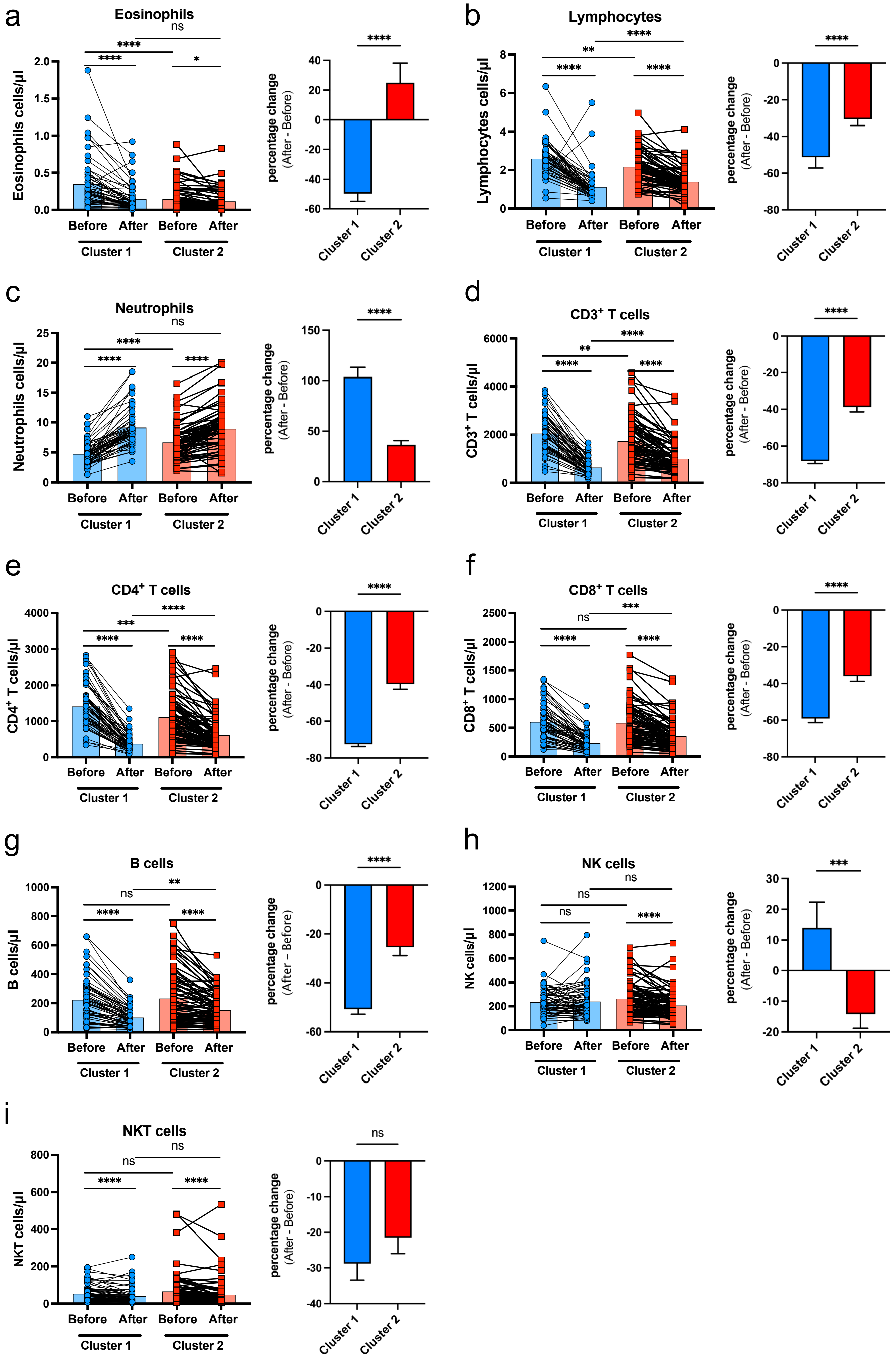

Supplementary Fig.1 Cluster 1 and 2 patients with severe asthma differ at baseline in the absolute numbers of eosinophils, lymphocytes, neutrophils, CD3$^+$ T cells and CD4$^+$ T cells, as well as in the response of several cellular populations to intravenous corticosteroids administration. For both patient clusters, the absolute number before and after the intravenous administration of CS and the corresponding percentage change are shown for eosinophils **(a)**, lymphocytes **(b)**, neutrophils **(c)**, CD3$^+$ T cells **(d)**, CD4$^+$ T cells **(e)**, CD8$^+$ T cells **(f)**, B cells **(g)**, NK cells **(h)**, NKT cells **(i)**. The difference between paired samples was analyzed with the Wilcoxon signed rank test and the difference between groups with the Mann-Whitney test. **: p<0.01, ***: p<0.001, ****: p<0.0001, ns: not significant. Dots represent individual patients. Bars represent the mean values. Percentage changes are reported as means ± SEM.
